# Sex Differences in Hospitalizations in the 6 Months Before a Diagnosis of Transthyretin Amyloid Cardiomyopathy

**DOI:** 10.1016/j.jacadv.2026.102627

**Published:** 2026-03-25

**Authors:** Chun Shing Kwok, Adnan I. Qureshi, Richard P. Steeds, Gregg C. Fonarow, Biykem Bozkurt, Marianna Fontana, Julian D. Gillmore, William E. Moody

**Affiliations:** a Department of Cardiology, Mid Cheshire Hospitals NHS Foundation Trust, Crewe, United Kingdom; b Zeenat Qureshi Stroke Institute, University of Missouri, Columbia, Missouri, USA; c Institute of Cardiovascular Sciences, College of Medical and Dental Sciences, University of Birmingham, Birmingham, United Kingdom; d Department of Cardiology, Queen Elizabeth Hospital, University Hospital of Birmingham NHS Foundation Trust, Birmingham, United Kingdom; e Division of Cardiology, Ahmanson-UCLA Cardiomyopathy Center, University of California Los Angeles, Los Angeles, California, USA; f Cardiology Section, Winters Center for Heart Failure, Baylor College of Medicine, Houston, Texas, USA; g National Amyloidosis Centre, University College London, London, United Kingdom

**Keywords:** hospitalization, hypertensive heart disease, mortality, transthyretin amyloid cardiomyopathy

## Abstract

**Background:**

The influence of sex on the extent of missed opportunities for early diagnosis of transthyretin amyloid cardiomyopathy (ATTR-CM) has not been characterized.

**Objectives:**

The objective of the study was to determine the rate and causes of hospitalization preceding the index admission with ATTR-CM, stratifying analyses according to sex.

**Methods:**

We conducted a retrospective cohort study of the National Readmissions Database to evaluate the 6-month hospitalizations preceding an index admission with a diagnosis of ATTR-CM.

**Results:**

Between 2018 and 2020, there were 10,975 patients hospitalized with ATTR-CM of whom 4,545 (41.1%) had 1 or more hospital admission within the preceding 6 months. Factors associated with an increased risk of previous admission included female sex (OR: 1.39; 95% CI: 1.14-1.69; *P* = 0.001) and hypertension (OR: 1.40; 95% CI: 1.04-1.88; *P* = 0.026). Females hospitalized with ATTR-CM were more likely to have a lower financial income based on zip code quartiles. The most common primary diagnoses for the preceding 6-month admissions before hospitalization with ATTR-CM were hypertensive heart disease with or without renal disease (32.5%), sepsis (4.9%), atrial fibrillation/flutter (4.1%), and heart failure (3.6%). A previous 6-month hospitalization was associated with a 2-fold increased risk of mortality during the index admission with ATTR-CM (OR: 2.06; 95% CI: 1.32-3.20; *P* = 0.001).

**Conclusions:**

Hospitalization occurs in nearly half of all patients in the 6 months before their admission with a diagnosis of ATTR-CM. The most common reasons for prior hospitalization include hypertensive heart and chronic kidney disease, sepsis, atrial arrhythmia, and heart failure. Previous hospitalization is more common among females and in the overall population is associated with a 2-fold increase in mortality.

Transthyretin (TTR) is a tetrameric protein that participates in the plasma transport of both thyroxine and retinol. It is primarily synthesized by the liver and disease occurs when it dissociates into oligomers and monomers that subsequently misfold into pathogenic transthyretin amyloid insoluble fibrils. Transthyretin amyloid cardiomyopathy (ATTR-CM) is caused by extracellular TTR-derived insoluble fibrils depositing in the myocardium, which results in a restrictive cardiomyopathy causing progressive heart failure that often proves fatal.^1^ Advances in diagnostic modalities and therapeutic options, have resulted in the increased detection of ATTR-CM and there are several classes of disease-modifying therapy either available or under active investigation.^2^ Despite recognized red flags for cardiac amyloidosis,^3^ delays to diagnosis of cardiac amyloidosis have been reported in several studies from the United Kingdom,^4^ Denmark,^5^ United States,^6^ and Germany.^7^ Cardiac amyloidosis is underdiagnosed, particularly in females,^8^ and timely diagnosis of ATTR-CM is crucial as there may only be a small window of opportunity where patients can derive benefit from novel treatments, beyond which therapies are less effective.^9^

Missed opportunities refer to incidences where different actions could have resulted in more favorable outcomes.^10^ Considering a pathway model for disease, patients begin in a healthy state before disease develops which at some point may manifest in the presence of symptoms and signs and consequential health care contacts.^11^ These health care contacts include hospital admissions but a key aspect is that patients decide to seek help. Patients with heart failure, a frequent presentation for patients with underlying ATTR-CM, may have potential missed opportunities related to diagnosis that are a consequence of patient, clinician, and health economy–related factors.^12^ Even if the presence of a heart failure syndrome is correctly identified, there may be missed opportunities related to treatment which should encompass a targeted disease-modifying therapy as well as conventional heart failure and device therapies and potential access to transplant and palliative care.^13^ Recently, a new methodology was developed to evaluate hospital admissions before a hospitalization of interest,^14^ with the aim of identifying potential missed opportunities for earlier diagnosis.

Little is known about the influence of sex on hospital admissions before the diagnosis of ATTR-CM. In this study, using data from the National Readmission Database (NRD), the primary aim was to establish the cause and frequency of hospitalizations in the 6-month period before the index admission with ATTR-CM and to assess for any differences observed between sexes. Our secondary objectives were to determine the factors associated with in-hospital all-cause mortality and hospital length of stay.

## Methods

### Study design

This study is a retrospective cohort study of the NRD data set. The NRD has an estimated 32.9 million hospital discharges each year and is a representative of all payers in the United States.^15^ It is a data set produced by the American Agency for Healthcare Research and Quality as a part of the Healthcare Cost and Utilization Project, designed to support various types of analyses of national readmissions which contains more than 100 clinical and nonclinical variables for each hospital stay. The methodology for the current study is based on a previous study, which evaluated 30-day previous admissions before ST-elevation myocardial infarction.^14^ This study was performed in accordance with the Strengthening the Reporting of Observational Studies in Epidemiology (STROBE) criteria.^16^ Ethical approval is not required for analyses of data sets produced by the Healthcare Cost and Utilization Project.^17^

### Study population

**Figure 1:**
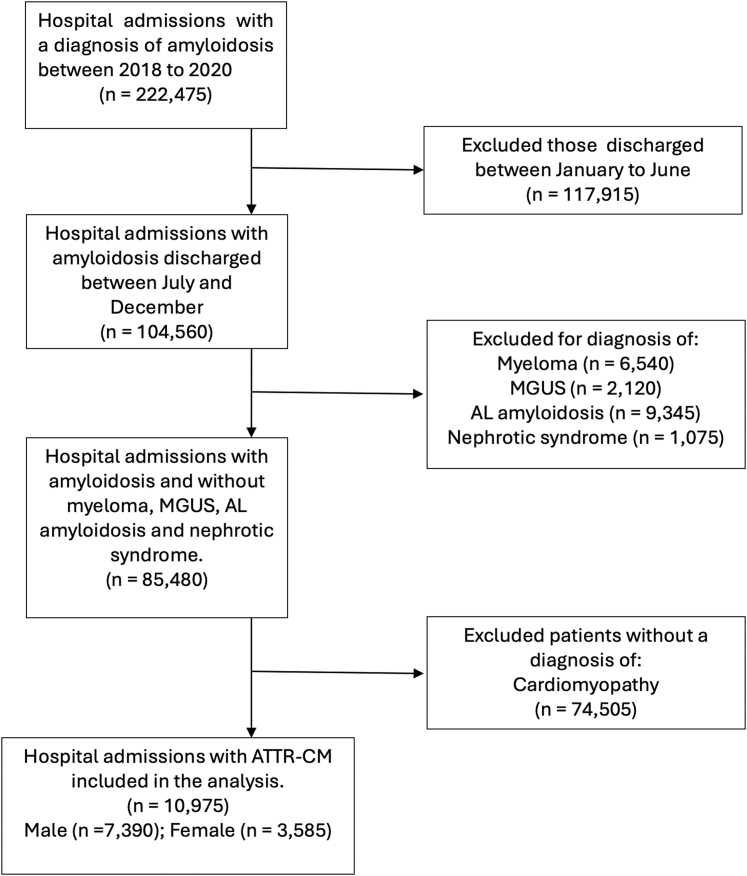
**Schematic Flow Diagram Detailing Identification of Hospitalizations for Transthyretin Amyloid Cardiomyopathy** After exclusions, there were a total of 10,975 hospitalizations included in the study among patients with ATTR-CM. AL = amyloid light chain; ATTR-CM = transthyretin amyloid cardiomyopathy; MGUS = monoclonal gammopathy of uncertain clinical significance.

Patients admitted to hospital with ATTR-CM between 2018 to 2020 were identified for inclusion in this study using International Classification of Diseases Tenth Revision (ICD-10) diagnostic coding (Figure 1). Patients with a hospital admission associated with an ICD-10 code of amyloidosis (E85∗) and cardiomyopathy (other hypertrophic cardiomyopathy I42.2, other restrictive cardiomyopathy I42.5, other cardiomyopathies I42.8, unspecified cardiomyopathy I42.9, and cardiomyopathy in metabolic disease I43.1) were included. To minimize the risk of including patients with a diagnosis of amyloid light chain cardiac amyloidosis, patients coded for that specific diagnosis amyloidosis (E85.81) and those with multiple myeloma (C90.0), monoclonal gammopathy of unknown significance (D47.2), and nephrotic syndrome (N04∗) were all excluded. We also excluded patients with missing values for age, sex and in-hospital outcome. Finally, patients admitted in the months of January to June were excluded because they would not have 6-month follow-up due to the nonlinkable annualized data sets in the NRD. Patients were categorized into 2 groups: those with and those without at least 1 hospital admission within the 6 months before their hospitalization with a diagnosis of ATTR-CM.

### Variables

We defined the characteristics of the patients based on variables available in the NRD and those defined by ICD-10 diagnostic codes for the first hospital admission as shown in Supplemental Table 1. The variables included in the analyses were age, sex, elective admission, weekend admission, primary expected payer, income quartile based on zip code, rural hospital admission, hospital bed size, hospital teaching status, pericardial effusion, atrial fibrillation, heart block, postural hypotension, aortic stenosis, neuropathy, smoking (nicotine dependence), alcohol misuse, hypertension, hypercholesterolemia, obesity, diabetes mellitus, previous myocardial infarction, heart failure, previous stroke, peripheral vascular disease, liver failure, chronic kidney disease (CKD), chronic lung disease, cancer, dementia, and palliative care. The cause of previous admissions was defined by the first ICD-10 code for the hospital admission based on the first 3 digits of the ICD-10 code.

### Statistical analysis

The statistical analyses were performed using Stata 13.0 (StataCorp LLC). The chi-square test was used for comparisons of categorical variables and the Wilcoxon rank-sum test was used for the comparison of continuous variables. Multivariable logistic regression models with backward selection were used to identify predictors of hospitalization in the 6 months before readmission with ATTR-CM and subsequent in-hospital mortality, as well as to identify factors independently associated with female sex among patients with ATTR-CM. Models were constructed across all patients and with analyses stratified by sex. The variables considered in the model included age, sex, elective admission, weekend admission, primary expected payer, income quartile based on zip code, rural hospital, hospital bed size, teaching hospital, pericardial effusion, atrial fibrillation, heart block, postural hypotension, aortic stenosis, neuropathy, smoking, alcohol misuse, hypertension, hypercholesterolemia, obesity, diabetes mellitus, previous heart attack, heart failure, previous stroke, peripheral vascular disease, liver failure, chronic lung disease, cancer, dementia, and palliative care. Results are presented as the OR with 95% CI. To allow for interpretation of sex differences, we also constructed additional models to evaluate the interaction terms for sex and covariates including age, hypertension, diabetes mellitus, obesity, CKD, and palliative care. A *P* value of <0.05 was considered statistically significant.

## Results

**Figure 2:**
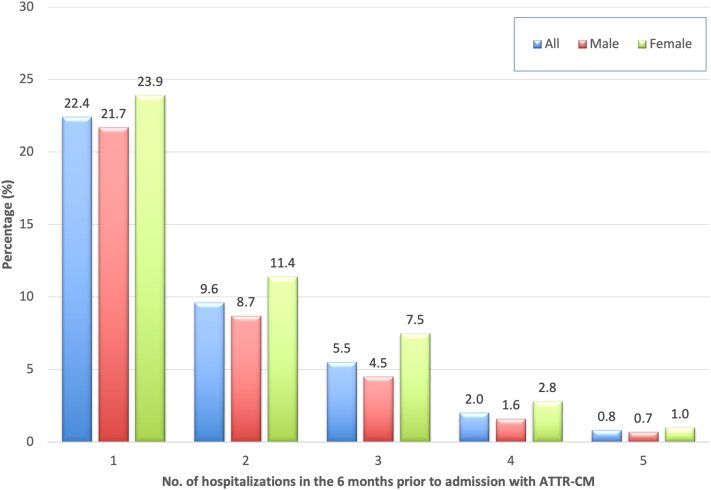
**Percentage Frequency of Hospitalizations Preceding Diagnosis of Transthyretin Amyloid Cardiomyopathyby Sex** Frequency of hospitalizations according to sex in the 6 months before readmission with a diagnosis of transthyretin amyloid cardiomyopathy (n = 10,975). Abbreviation as in Figure 1.

There were 10,975 patients with ATTR-CM included in the study and a total of 15,520 hospital admissions within the overall study period. At least 1 hospital admission within 6 months before the index hospitalization with ATTR-CM occurred in 4,545 (41.4%) of patients (Figure 2) and the proportion of patients with 2, 3, 4, and 5 admissions within the preceding 6-month period was 19.0%, 3.9%, 1.9%, and 1.1%, respectively.

### Characteristics of patients hospitalized with ATTR-CM according to sex

The demographics and characteristics of patients included in the study stratified by sex are displayed in Table 1. Females hospitalized with ATTR-CM were more likely to require a nonelective or weekend admission and have a lower financial income based on zip code quartiles. A similarly high proportion of males and females had a background of hypertension (86.1% male vs 88.2% female; *P* = 0.18). In contrast, females had higher rates of obesity and dementia but were less likely to have been diagnosed with atrial arrhythmia, heart block, and CKD. There were no sex differences in the hospital length of stay or costs associated with the index admission for ATTR-CM. A significantly higher proportion of females were hospitalized in the 6-month period before their diagnosis of ATTR-CM (47.4% females vs 38.5% males; *P* < 0.001).

**Table 1:** Characteristics of Patients Hospitalized With Transthyretin Amyloid Cardiomyopathy According to Sex (N = 10,975)

	Male (n = 7,390)	Female (n = 3,585)	*P* Value
Age (y)	75 [65 to 83]	75 [66 to 83]	0.49
Elective admission	9.1	5.3%	0.002
Weekend admission	18.9	23.2%	0.019
Primary expected payer			0.028
Medicare	76.0%	80.6%	
Medicaid	7.1%	7.7%	
Private insurance	12.6%	9.1%	
Self-pay	1.6%	1.5%	
No charge	0.2%	0.1%	
Other	2.5%	1.0%	
Zip income quartile			<0.001
0th-25th	23.6%	31.1%	
26th-50th	23.6%	25.0%	
51st-75th	24.8%	22.2%	
76th-100th	28.0%	21.7%	
Rural hospital	10.6%	6.9%	0.005
Hospital bed size			0.063
Small	13.4%	17.0%	
Medium	26.5%	26.6%	
Large	60.2%	56.4%	
Teaching hospital	85.8%	86.2%	0.80
Pericardial effusion	4.3%	7.5%	0.002
Atrial fibrillation and atrial flutter	57.9%	52.4%	0.015
Heart block	7.5%	5.0%	0.029
Postural hypotension	2.6%	2.4%	0.71
Aortic stenosis	4.1%	5.6%	0.13
Neuropathy	5.1%	4.6%	0.63
Smoking	0.3%	0.7%	0.24
Alcohol misuse	1.4%	0.4%	0.044
Hypertension	86.1%	88.2%	0.18
Hypercholesterolemia	55.3%	47.7%	0.001
Obesity	12.3%	15.9%	0.021
Diabetes mellitus	31.7%	35.0%	0.12
Previous myocardial infarction	8.9%	9.2%	0.79
Heart failure	81.7%	80.8%	0.58
Previous stroke	15.2%	16.7%	0.36
Peripheral vascular disease	3.7%	4.3%	0.45
Liver failure	1.4%	0.8%	0.24
Chronic kidney disease	57.0%	49.9%	0.002
Chronic lung disease	21.2%	25.9%	0.013
Cancer	6.6%	5.0%	0.14
Dementia	8.1%	11.0%	0.027
Palliative care	4.1%	4.3%	0.83
Death on admission for ATTR-CM	5.0%	5.3%	0.77
Length of stay for admission with amyloidosis (days)	6 [3-11]	6 [3-10]	0.72
Cost for admission with amyloidosis ($)	16,160 [8,728 to 32,630]	14,750 [8,691 to 27,445]	0.13
Previous hospitalization within 6-months	38.5%	47.4%	<0.001

Values are median (25th-75th percentiles) or n (%) and compared using Wilcoxon rank-sum test or chi-square test, respectively.

ATTR-CM = transthyretin amyloid cardiomyopathy.

The independent factors associated with female sex among this cohort of patients with ATTR-CM are displayed in Supplemental Table 2. The presence of pericardial effusion (OR: 1.82; 95% CI: 1.22-2.72; *P* = 0.003) and obesity (OR: 1.56; 95% CI: 1.18-2.07; *P* = 0.002) were significantly associated with being female although females had lower odds of having a prior diagnosis of heart block (OR: 0.65; 95% CI: 0.43-0.98; *P* = 0.037). Females also had lower odds of being within the highest quartile of income (OR: 0.50; 95% CI: 0.38-0.80; *P* < 0.001), and a lower odds of being electively admitted (OR: 0.64; 95% CI: 0.44-0.95; *P* = 0.027) or having private insurance (OR: 0.64; 95% CI: 0.46-0.90; *P* = 0.011).

### Characteristics of patients with and without a prior 6-month hospital admission

Table 2 shows the characteristics of the patients stratified according to any previous admission or no previous admission within the 6 months of the index hospitalization with ATTR-CM. Patients with 1 or more prior admission were younger (median age 73 [Q1-Q3: 63-82] vs 77 [Q1-Q3: 68-83] years, *P* < 0.001) and had a higher prevalence of hypertension (90.8% vs 83.9%; *P* < 0.001), CKD (59.4% vs 51.4%; *P* < 0.001), obesity (16.9% vs 11.0%; *P* < 0.001), and diabetes mellitus (38.9% vs 28.4%; *P* < 0.001). In contrast, those with a prior admission had a lower prevalence of pericardial effusion (4.1% vs 6.3%, *P* = 0.027) and neuropathy (2.6% vs 6.5%, *P* < 0.001). The rate of in-hospital mortality was 5.8% for patients with previous admissions compared to 4.6% for those with no admission within 6 months (*P* = 0.20). The median length of stay for the index admission with ATTR-CM was 7 days (Q1-Q3: 4-12) for those patients with a previous 6-month hospitalization vs 5 days (Q1-Q3: 3-10) in those without a prior admission (*P* < 0.001). There was no significant difference in cost for the index hospital admission with ATTR-CM between those patients with and without a previous 6-month admission ($16,343 vs $15,257, *P* = 0.25).

**Table 2:** Characteristics of Patients With and Without Hospitalization in the 6 months Before Readmission With Transthyretin Cardiac Amyloidosis

	No Previous Hospitalization in the Preceding 6 Months (n = 6,430)	Previous Hospitalization in the Preceding 6 Months (n = 4,545)	*P* Value
Age (y)	77 [68-83]	73 [63-82]	<0.001
Female	1,884 (29.3)	1,700 (37.4)	<0.001
Elective admission	540 (8.4)	327 (7.2)	0.29
Weekend admission	124 (19.3)	986 (21.7)	0.17
Primary expected payer			0.001
Medicare	4,990 (77.6)	3,522 (77.5)	
Medicaid	354 (5.5)	450 (9.9)	
Private insurance	823 (12.8)	436 (9.6)	
Self-pay	109 (1.7)	59 (1.3)	
No charge	13 (0.2)	5 (0.1)	
Other	148 (2.3)	77 (1.7)	
Zip income quartile			<0.001
0th-25th	1,485 (23.1)	1,373 (30.2)	
26th-50th	1,505 (23.4)	1,136 (25.0)	
51st-75th	1,569 (24.4)	1,059 (23.3)	
76th-100th	1878 (29.2)	973 (21.4)	
Rural hospital	604 (9.4)	427 (9.4)	0.99
Hospital bed size			<0.001
Small	778 (12.1)	827 (18.2)	
Medium	1768 (27.5)	1,145 (25.2)	
Large	3,890 (60.5)	2,577 (56.7)	
Teaching hospital	5,658 (88.0)	3,772 (83.0)	0.001
Pericardial effusion	405 (6.3)	186 (4.1)	0.023
Atrial fibrillation and atrial flutter	3,704 (57.6)	2,454 (54.0)	0.094
Heart block	476 (7.4)	259 (5.7)	0.12
Postural hypotension	206 (3.2)	77 (1.7)	0.024
Aortic stenosis	283 (4.4)	218 (4.8)	0.65
Neuropathy	418 (6.5)	118 (2.6)	<0.001
Alcohol misuse	71 (1.1)	45 (1.0)	0.82
Hypertension	5,395 (83.9)	4,127 (90.8)	<0.001
Hypercholesterolemia	3,234 (50.3)	2,559 (56.3)	0.005
Obesity	707 (11.0)	768 (16.9)	<0.001
Diabetes mellitus	1826 (28.4)	1768 (38.9)	<0.001
Previous myocardial infarction	527 (8.2)	459 (10.1)	0.11
Heart failure	5,260 (81.8)	3,677 (80.9)	0.58
Previous stroke	932 (14.5)	795 (17.5)	0.055
Peripheral vascular disease	231 (3.6)	195 (4.3)	0.39
Liver failure	90 (1.4)	45 (1.0)	0.39
Chronic kidney disease	3,305 (51.4)	2,700 (59.4)	<0.001
Chronic lung disease	1,228 (19.1)	1,264 (27.8)	<0.001
Cancer	444 (6.9)	227 (5.0)	0.058
Dementia	611 (9.5)	386 (8.5)	0.41
Palliative care	399 (6.2)	59 (1.3)	<0.001
Death on admission for ATTR-CM	296 (4.6)	264 (5.8)	0.19
Length of stay for admission with amyloidosis (days)	5 [3-10]	7 [4-12]	<0.001
Cost for admission with amyloidosis ($)	15,257 [8,489-29,503]	16,343 [9,189-31,929]	0.074

Values are median (25th-75th percentiles) or n (%) and compared using Wilcoxon rank-sum test or chi-square test, respectively.

Abbreviation as in Table 1.

### Causes of 6-month previous admissions

**Figure 3:**
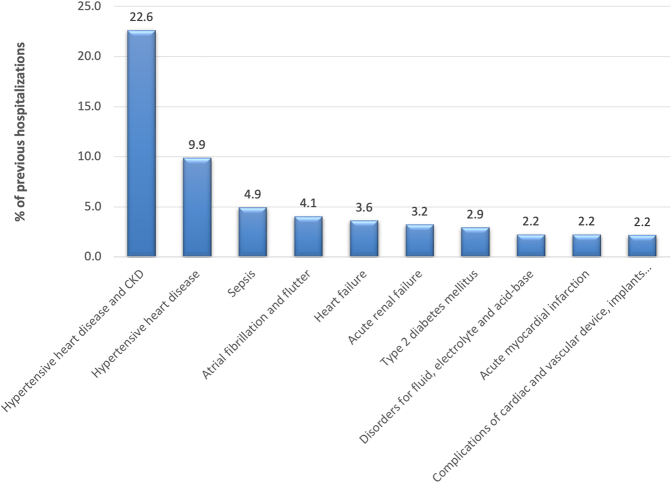
**Top 10 Primary Diagnoses Accounting for Hospitalization Preceding Transthyretin Amyloid CardiomyopathyDiagnosis** Top 10 primary diagnoses in the 6 months before the index admission with a diagnosis of transthyretin amyloid cardiomyopathy (n = 4,545). The primary diagnosis was assigned according to International Classification of Diseases-10th Revision diagnostic coding. CKD = chronic kidney disease.

The most common diagnoses for the 6-month admissions before hospitalization with ATTR-CM are shown in Figure 3. Hypertensive heart disease with or without renal disease accounted for 32.5% of all primary diagnoses for the previous admissions. There were also previous admissions for sepsis (4.9%), atrial fibrillation and flutter (4.1%), and heart failure (3.6%).

### Factors associated with previous 6-month admissions

The factors most strongly associated with a previous 6-month admission before hospitalization with ATTR-CM were female sex (OR: 1.39; 95% CI: 1.14-1.69; *P* = 0.001), chronic lung disease (OR: 1.56; 95% CI: 1.26-1.94; *P* < 0.001), hypertension (OR: 1.40; 95% CI: 1.04-1.88; *P* = 0.026), obesity (OR: 1.33; 95% CI: 1.01-1.75; *P* = 0.043), and diabetes mellitus (OR: 1.31; 95% CI: 1.07-1.60; *P* = 0.009) (Table 3). The results of formal interaction testing between sex and covariates are displayed in Supplemental Table 3. There were reduced odds of previous admission for patients with private insurance (OR: 0.60; 95% CI: 0.43-0.83; *P* = 0.005) or in those treated in large vs small hospitals (OR: 0.58; 95% CI: 0.44-0.75; *P* < 0.001), as well as those having concomitant neuropathy (OR: 0.43; 95% CI: 0.26-0.70; *P* = 0.001).

**Table 3:** Multivariable Logistic Regression to Identify Predictors of Hospitalization in the 6 Months Before Readmission With Transthyretin Amyloid Cardiomyopathy According to Sex

	Male		Female		Total	
	OR (95% CI)	*P* Value	OR (95% CI)	*P* Value	OR (95% CI)	*P* Value
Age	0.98 (0.97-0.99)	0.005	0.97 (0.96-0.99)	0.001	0.98 (0.97-0.99)	<0.001
Female	-	-	-	-	1.39 (1.14-1.69)	0.001
Primary expected payer vs Medicare						
Private insurance	0.64 (0.43-0.93)	0.021	0.50 (0.27-0.95)	0.034	0.60 (0.43-0.83)	0.002
Self-pay	-	-	0.05 (0.01-0.48)	0.009	-	-
Zip income quartile vs 0th to 25th income quartile						
51st to 75th income quartile	0.63 (0.46-0.88)	0.007	-	-	-	-
76th to 100th income quartile	0.56 (0.40-0.78)	0.001	-	-	0.68 (0.52-0.89)	0.005
Hospital size vs small						
Medium	0.43 (0.29-0.62)	<0.001	-	-	0.53 (0.40-0.72)	<0.001
Large	0.54 (0.39-0.76)	<0.001	0.61 (0.38-0.96)	0.034	0.58 (0.44-0.75)	<0.001
Teaching hospital	0.69 (0.50-0.94)	0.020	0.49 (0.30-0.81)	0.005	0.62 (0.48-0.80)	<0.001
Pericardial effusion	-	-	0.46 (0.24-0.88)	0.019	0.58 (0.38-0.89)	0.013
Neuropathy	0.45 (0.24-0.83)	0.011	0.37 (0.15-0.89)	0.027	0.43 (0.26-0.70)	0.001
Hypertension	-	-	1.87 (1.08-3.22)	0.025	1.40 (1.04-1.88)	0.026
Hypercholesterolemia	1.31 (1.03-1.66)	0.030	-	-	1.28 (1.06-1.55)	0.001
Obesity	1.81 (1.01-3.27)	0.001	-	-	1.33 (1.01-1.75)	0.043
Diabetes mellitus	-	-	1.59 (1.11-2.29)	0.011	1.31 (1.07-1.60)	0.009
Peripheral vascular disease	1.81 (1.01-3.27)	0.048	-	-	-	-
Chronic kidney disease	1.30 (1.03-1.65)	0.030	-	-	1.28 (1.05-1.54)	0.013
Chronic lung disease	1.41 (1.07-1.86)	0.014	1.72 (1.18-2.52)	0.005	1.56 (1.26-1.94)	<0.001
Palliative care	0.22 (0.10-0.51)	<0.001	0.22 (0.08-0.60)	0.003	0.23 (0.12-0.44)	<0.001

### Factors associated with in-hospital mortality

Previous hospitalization was associated with increased odds of in-hospital all-cause mortality on the index admission with ATTR-CM (OR: 2.06; 95% CI: 1.32-3.20; *P* = 0.001) (Table 4). The strongest predictors of mortality were palliative care input (OR: 11.05; 95% CI: 6.13-19.94; *P* < 0.001), liver failure (OR: 8.96; 95% CI: 3.46-23.21; *P* < 0.001), and cancer (OR: 2.59; 95% CI: 1.35-4.96; *P* = 0.004). The presence of hypertension was associated with reduced mortality (OR: 0.47; 95% CI: 0.27-0.82; *P* = 0.008). The results of formal interaction testing between sex and covariates are displayed in Supplemental Table 4.

**Table 4:** Multivariable Logistic Regression to Identify Predictors of In-Hospital Mortality During Hospitalization With Transthyretin Amyloid Cardiomyopathy According to Sex

	Men		Women		Total	
	OR (95% CI)	*P* Value	OR (95% CI)	*P* Value	OR (95% CI)	*P* Value
Previous hospitalization within 6-months	2.52 (1.45-4.41)	0.001	1.33 (0.60-2.93)	0.48	2.06 (1.32-3.20)	0.001
Hypertension	0.46 (0.24-0.88)	0.018	-	-	0.47 (0.27-0.82)	0.008
Liver failure	8.57 (2.61-28.11)	<0.001	11.94 (1.37-104.21)	0.025	8.96 (3.46-23.21)	<0.001
Cancer	-	-	4.02 (1.19-13.59)	0.025	2.59 (1.35-4.96)	0.004
Palliative care	15.30 (7.39-31.68)	<0.001	5.65 (1.74-18.37)	0.004	11.05 (6.13-19.94)	<0.001

## Discussion

To our knowledge, this is the first study using data from the NRD to examine antecedent factors associated with a subsequent admission for a new diagnosis of ATTR-CM. There are several key findings, which offer novel insight into the care pathways for patients with ATTR-CM (Central Illustration). First, we demonstrate that nearly half (41.1%) of patients hospitalized with ATTR-CM are admitted to hospital in the preceding 6 months and more than a quarter (25.9%) require 2 or more admissions within that same period. Second, there was a higher risk of hospitalization before the diagnosis of ATTR-CM among females and among individuals of lower socioeconomic status. Third, a substantial proportion of patients with ATTR-CM (31.0%) appear to be previously and in retrospect, mislabeled with a diagnosis of hypertensive heart disease (with or without renal involvement), as the primary cause for preceding hospital admissions. Other common diagnoses associated with admission included sepsis, heart failure and atrial fibrillation or flutter. Finally, an admission in the preceding 6 months was associated with a 2-fold increase in in-hospital mortality during the readmission with ATTR-CM and a longer hospital length of stay.

**Central Illustration:**
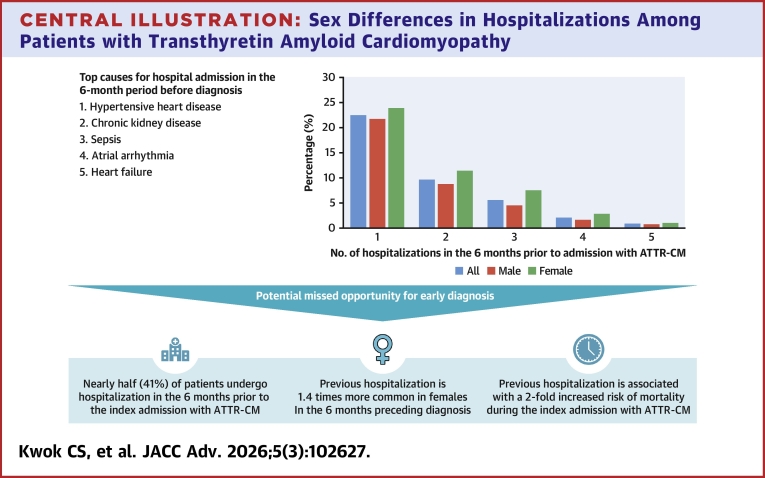
**Sex Differences in Hospitalizations Among Patients with Transthyretin Amyloid Cardiomyopathy** Hospitalizations among patients with transthyretin amyloid cardiomyopathy in the 6 months before diagnosis (N = 10,975). ATTR-CM = transthyretin amyloid cardiomyopathy.

Although numerous studies have highlighted the historical delays in diagnosis of ATTR-CM, there is a paucity of data examining whether delay in diagnosis is more frequent among females. To our knowledge, this is the first large-scale study to discover sex differences in the frequency and cause of hospital admissions before a diagnosis of ATTR-CM is made. We have demonstrated important sex-related differences in hospital admission on a national level: there was a 1.4-fold increased risk of hospitalization among females in the 6 months before their subsequent diagnosis of ATTR-CM. Moreover, females affected by ATTR-CM are at a higher risk of recurrent hospital admissions in the 6-month period before the diagnosis is clinched. These findings support the notion that because there is less clinical suspicion for ATTR-CM in females, they have greater potential to initially be misdiagnosed and, therefore, present with more advanced stages of disease.

Although a deep phenotyping study of 1,732 patients with ATTR-CM suggested no overall differences between sexes in clinical phenotype with respect to disease progression and prognosis,^8^ 2 more recent reports have suggested females are less frequently diagnosed, older at presentation, and more likely to present with a history of heart failure and/or carpal tunnel syndrome.^18^^,^^19^ In the present study, although there was no difference in age at diagnosis between sexes, females were less likely to have a history of heart block and atrial arrhythmia; the absence of such red flags could conceivably contribute to lowering the clinical suspicion for ATTR-CM. In keeping with our findings, which have demonstrated the potential for delays in diagnosis to affect females more than males, a recent study suggested females are more likely to present with markers of advanced disease such as worse renal function and higher filling pressures.^19^ In contrast, in a survey from the Transthyretin Amyloidosis Outcomes Survey of 2,790 patients, myocardial involvement was more frequent and pronounced in male patients with hereditary ATTR-CM, suggesting sex-related biological characteristics that may inhibit myocardial amyloid infiltration.^20^

It is possible that the repeated admissions seen in a quarter of patients within the 6-months before a subsequent diagnosis of ATTR-CM is made represent delays in diagnosis and a missed opportunity to begin timely treatment. The emergence of novel disease-modifying therapies has transformed ATTR-CM from being a condition that was regarded as largely palliative to one where early diagnosis and treatment significantly improves prognosis.^16^^,^^21^ In response to this change in mindset, compared with 20 years ago, there has been a more than 27-fold increase in diagnosis of ATTR-CM in the last 5 years.^22^ Nevertheless, many patients are still diagnosed late. It has been reported that patients use hospital services a median of 17 times during the 3 years before diagnosis.^23^ A median diagnostic delay of 3 years for ATTR-CM is currently unacceptable in the knowledge that early treatment with TTR targeted therapy offers the most favorable outcome.^24^ With early diagnosis proving critical, considering the cause for hospitalization before diagnosis offers an opportunity to better characterize the patient diagnostic journey.

Echocardiography has an important role as a screening tool for patients in whom there is suspicion of cardiac amyloidosis,^25^ but based on this study, not all primary diagnoses will lead to a request for cardiac imaging. Although patients with hypertensive heart disease, heart failure, atrial fibrillation and flutter may undergo echocardiography, those presenting with sepsis, and acute renal failure will often not undergo cardiac imaging. In the current study, the most common primary diagnosis for admissions in the 6 months before a hospitalization with ATTR-CM was “hypertensive heart disease with or without kidney disease.” This finding suggests that many patients with ATTR-CM continue to be misdiagnosed, perhaps because of a tendency for left ventricular hypertrophy to be attributed to hypertensive heart disease, either in isolation or in association with CKD. Our study supports the notion that a finding of unexplained left ventricular hypertrophy should always raise a suspicion of cardiac amyloidosis.^26^

Hypertensive heart disease refers to the constellation of changes in the left ventricle, left atrium, and coronary arteries as a consequence of chronic blood pressure elevation which increases workload on the heart resulting in adverse structural and functional changes.^27^ Left ventricular hypertrophy affects 20% of patients with mild hypertension and is almost universal in patients with severe or complicated hypertension,^28^ and an independent risk factor for cardiovascular morbidity and mortality.^29^ As hypertension affects almost 1 in 3 adults in the world,^30^ it is not unreasonable when left ventricular hypertrophy is detected to make a diagnosis of hypertensive heart disease. Many patients are ultimately diagnosed with ATTR-CM on a background of hypertension and the 2 pathologies often coexist. In the current study as many as 91% of patients admitted in the 6 months before diagnosis with ATTR-CM had a historical diagnosis of hypertension. Although a reduced requirement for antihypertensive therapy is an established red flag for ATTR-CM, this can be a late sign; those patients with less advanced disease will often still exhibit hypertension. Furthermore, cardiac amyloidosis is a condition notorious for mimicking other diseases with a number of hypertrophic phenocopies.^31^ In particular, the echo findings of ATTR-CM are not highly specific which can lead to misdiagnosis of phenocopies such as hypertensive heart disease and hypertrophic cardiomyopathy.^32^ Although unexplained left ventricular hypertrophy should raise a suspicion of cardiac amyloidosis,^26^ not all centers will routinely examine patients for apical sparing of longitudinal strain. Advanced multimodality imaging with bone scintigraphy and cardiac magnetic resonance imaging provide complementary utility and offer much greater diagnostic specificity when differentiating between hypertrophic phenotypes.

Our study adds to the growing evidence that socioeconomic status influences outcomes among patients with ATTR-CM, after demonstrating a higher risk of hospitalization in the 6-months before diagnosis for those patients in the lowest quartile of income, and a lower risk among those with private health insurance. This finding could be explained by an inequity of access to advanced diagnostic imaging, leading to late diagnosis of ATTR-CM resulting in more admissions to hospital in association with more disease-related complications (heart failure, atrial arrhythmia). In an evaluation of 189 U.S. patients diagnosed with ATTR-CM, the majority were White males who spoke English as their primary language, whereas the median income was skewed toward upper income. Furthermore, there were notable delays in patient access to tafamidis among those requiring financial assistance.^33^ Another report assessing the impact of race and socioeconomic status among patients with ATTR-CM found that Black individuals of lower socioeconomic status were at a greater risk of underdiagnosis and adverse outcome compared to White patients.^34^ In keeping with the current study, high deprivation index has also been linked to delayed diagnosis of ATTR-CM and Black individuals are more likely to present with more advanced stages of disease.^35^

Although aortic stenosis often coexists in patients with ATTR cardiac amyloidosis and affected 4% of males and 6% of females in this cohort, it was not among the most common primary reasons for prior admission in the 6-month period before diagnosis of ATTR-CM. It is conceivable that it will have contributed to some admissions with heart failure. Aortic stenosis was in greater proportion as a comorbidity in the group with a prior admission in the 6 months preceding the ATTR-CM diagnosis (4.8% vs 4.4%), but this did not meet statistical significance. It is notable that the liver-disease rates were low in the overall cohort (1.2%), but the presence of liver disease was strongly linked to higher mortality, and this association was particularly robust in females.

We found that liver failure was the strongest factor associated with in-hospital mortality among this cohort of patients with ATTR-CM. Heart failure and liver disease often coexist and the latter may vary from acute cardiogenic liver injury to congestive hepatopathy.^36^ Hepatic dysfunction is commonly seen in patients with heart failure and closely correlated with a poor outcome.^37^ Data from the ASCEND-HF study reported that > 40% of patients hospitalized with acute heart failure had abnormal liver function tests and elevated bilirubin was associated with worse clinic outcome.^38^ The spectrum of heart and liver disease can be classified as heart disease affecting the liver, liver disease affecting the heart and conditions affecting the heart and liver.^39^ Hepatic amyloidosis is rare but it is recognized that amyloid protein may deposit in the liver.^40^ Multiorgan dysfunction is associated with poor prognosis in ATTR-CM as hyperbilirubinemia and increased alkaline phosphatase were associated with a 1.32- and 1.20-fold increase, respectively, in the risk of mortality among 2,566 patients with ATTR-CM.^41^ Our findings support the existing evidence that congestive hepatopathy portends a poor prognosis.

### Study Limitations

These data are observational and reliant on the quality of discharge summaries and coding. Residual measured and unmeasured confounding may influence these findings. An important limitation of the NRD is that each year represents an independent data set with an inability to link data across more than 1 calendar year. We therefore had to limit our prediagnosis surveillance window to 6 months to allow for the inclusion of a high number of patients with adequate follow-up. This study does not have information on ethnicity, medications and imaging, nor was there access to the results of TTR genetic testing and so we cannot make inferences regarding hereditary vs wild-type ATTR-CM. This work has deliberately focused on ATTR cardiac amyloidosis. It is unknown, however, whether similar sex differences might apply to patients diagnosed with amyloid light chain cardiac amyloidosis; this area should be targeted in future work. Finally, there was a lack of data available on prior pregnancies, the presence of pregnancy-related complications, and hormonal status.

## Conclusions

In this analysis of nationally representative hospital in-patient data from the United States, nearly half of patients (41.1%) with ATTR-CM had a previous hospitalization in the 6-months before diagnosis, while more than a quarter (25.9%) had 2 or more admissions. The most common primary causes assigned for the preceding admissions were hypertensive heart disease, sepsis, atrial arrhythmia, and heart failure. Females with ATTR-CM were more likely to be admitted in the 6-month period before diagnosis, and in the overall population, this was associated with a 2-fold increase in mortality on subsequent hospital admission.

## Funding support and author disclosures

Dr Kwok has received research grant support from 10.13039/100004319Pfizer and 10.13039/100004326Bayer for an unrelated project. Dr Steeds reports consultancy for 10.13039/100015362Amicus Therapeutics and 10.13039/100004325AstraZeneca. Dr Fonarow reports consultancy for 10.13039/100000046Abbott, 10.13039/100002429Amgen, 10.13039/100004325AstraZeneca, 10.13039/100004326Bayer, 10.13039/100001003Boehringer Ingelheim, 10.13039/100014941Cytokinetics, Eli Lilly, 10.13039/100004331Johnson & Johnson, 10.13039/100004374Medtronic, 10.13039/100004334Merck, 10.13039/100004336Novartis, and 10.13039/100004319Pfizer. Dr Fontana reports consultancy for 10.13039/100006396Alexion/Caelum Biosciences, 10.13039/100006400Alnylam, 10.13039/100004325AstraZeneca, Attralus, 10.13039/100004326Bayer, BridgeBio/Eidos, Cardior, Intellia Therapeutics, 10.13039/100013669Ionis Pharmaceuticals, 10.13039/100008897Janssen Pharmaceuticals, Lexeo Therapeutics, MyCardium AI, 10.13039/501100004191Novo Nordisk, 10.13039/100004319Pfizer, and 10.13039/100015364Prothena; research grants from 10.13039/100006400Alnylam, 10.13039/100004325AstraZeneca, BridgeBio, and 10.13039/100004319Pfizer; and she has share options in Lexeo Therapeutics and MyCardium AI. Dr Gillmore has done consulting for 10.13039/100006400Alnylam Pharmaceuticals, 10.13039/100004325AstraZeneca, Attralus, BridgeBio Pharma, Intellia Therapeutics, 10.13039/100013669Ionis Pharmaceuticals, Lycia Therapeutics, and 10.13039/100004319Pfizer. Dr Bozkurt reports consultancy for 10.13039/100000046Abbott, ABIOMED/10.13039/100004331Johnson and Johnson, 10.13039/100004326Bayer, 10.13039/100001003Boehringer Ingelheim, Cardurion, Cytokinetics, Eli Lilly, 10.13039/100004374Medtronic, 10.13039/100004334Merck, Idorsia, 10.13039/501100004191Novo Nordisk, Regeneron, Renovacor, Roche, Salubris, Sanofi-Aventis, scPharmaceuticals, Vifor, and Respicardia/Zoll. Dr Moody reports consultancy/speaker fees for 10.13039/100006400Alnylam, 10.13039/100004325AstraZeneca, 10.13039/100004326Bayer, 10.13039/100001003Boehringer Ingelheim, Bristol Myers Squibb, 10.13039/100013669Ionis Pharmaceuticals, 10.13039/100004319Pfizer, and Philips. All other authors have reported that they have no relationships relevant to the contents of this paper to disclose.
