# Supplementary material for: Sex Differences in Hospitalizations in the 6 Months Before a Diagnosis of Transthyretin Amyloid Cardiomyopathy
Source: JACC Adv. 2026 Mar 25;5(3):102627. doi: 10.1016/j.jacadv.2026.102627 (PMC13351999; doi:10.1016/j.jacadv.2026.102627)
Supplement: Supplemental Material [file mmc1.docx]

**Supplementary Table 1: Variables included in the analysis and data source**

| **Variable** | **Data source** |
| --- | --- |
| Amyloidosis | ICD-10 diagnosis code: E85 |
| Cardiomyopathy | ICD-10 diagnosis code: I42.2, I42.5, I42.8, K42.9, I43.1 |
| Cardiac amyloidosis | Amyloidosis and cardiomyopathy |
| Multiple myeloma | ICD-10 diagnosis code: C90.0 |
| Light-chain amyloidosis | ICD-10 diagnosis code: E85.81 |
| Monoclonal gammopathy of unknown significance | ICD-10 diagnosis code: D47.2 |
| Nephrotic syndrome | ICD-10 diagnosis code: N04 |
| Month discharge | AMONTH variable |
| Age | AGE variable |
| Female sex | FEMALE variable |
| Primary expected payer | PAY1 variable |
| ZIP income quartile | ZIPINC_QRTL variable |
| Rural hospital | PL_NCHS variable values 5 and 6 |
| Hospital Bed Size | HOSP_BEDSIZE variable |
| Teaching hospital | HOSP_UR_TEACH variable |
| Spinal stenosis | ICD-10 diagnosis code: M48.0 |
| Carpal tunnel syndrome | ICD-10 diagnosis code:G56.0 |
| Pericardial effusion | ICD-10 diagnosis code: I31.3 |
| Atrial fibrillation and flutter | ICD-10 diagnosis code: I48 |
| Heart block | ICD-10 diagnosis code: I44.0, I44.1, I44.2, I44.3 |
| Postural hypotension | ICD-10 diagnosis code: I95.1 |
| Aortic stenosis | ICD-10 diagnosis code: I35.0 |
| Neuropathy | ICD-10 diagnosis code: G60, G61, G62, G63, G64 |
| Smoking | ICD-10 diagnosis code: Z27.0 |
| Alcohol misuse | ICD-10 diagnosis code: F10.1 |
| Hypertension | ICD-10 diagnosis code: I10, I11, I12, I13, I15, I16 |
| Hypercholesterolemia | ICD-10 diagnosis code: E78.0, E78.1, E78.2, E78.3, E78.5 |
| Obesity | ICD-10 diagnosis code: E66.0, E66.1, E66.2, E66.8, E66.9 |
| Diabetes mellitus | ICD-10 diagnosis code: E08, E10, E11, E13 |
| Previous myocardial infarction | ICD-10 diagnosis code: I25.2 |
| Heart failure | ICD-10 diagnosis code: I09.81, I11.0, I50 |
| Previous stroke | ICD-10 diagnosis code: I69, Z86.73 |
| Peripheral vascular disease | ICD-10 diagnosis code: I73 |
| Liver failure | ICD-10 diagnosis code: K72 |
| Chronic kidney disease | ICD-10 diagnosis code: N18 |
| Chronic lung disease | ICD-10 diagnosis code: K72 |
| Cancer | ICD-10 diagnosis code: C* |
| Dementia | ICD-10 diagnosis code: F01, F02, G30, G31 |
| Palliative care | ICD-10 diagnosis code: Z51.5 |
| In-hospital mortality | DIED variable |
| Length of stay | LOS variable |
| In-hospital cost | TOTCHG x charge-to-cost ratio |

**Supplementary Table 2. Multivariable logistic regression to identify factors associated with female sex among patients with transthyretin cardiac amyloidosis**

| **Variable** | **Odds ratio (95%CI)** | **p-values** |
| --- | --- | --- |
| Elective | 0.64 (0.44-0.95) | 0.027 |
| Primary expected payer vs Medicare  Private insurance  Other | 0.64 (0.46-0.90)  0.35 (0.15-0.80) | 0.011  0.013 |
| ZIP income quartile vs 0^th^-25^th^  26^th^-50^th^  51^st^-75^th^  76^th^-100^th^ | 0.74 (0.57-0.96)  0.61 (0.46-0.79)  0.50 (0.38-0.80) | 0.024  <0.001  <0.001 |
| Rural hospital | 0.52 (0.36-0.74) | <0.001 |
| Hospital bed size vs small  Large | 0.71 (0.54-0.93) | 0.93 |
| Pericardial effusion | 1.82 (1.22-2.72) | 0.003 |
| Atrial fibrillation | 0.78 (0.64-0.96) | 0.019 |
| Heart block | 0.65 (0.43-0.98) | 0.037 |
| Hypercholesterolemia | 0.67 (0.55-0.82) | <0.001 |
| Obesity | 1.56 (1.18-2.07) | 0.002 |
| Chronic kidney disease | 0.70 (0.58-0.85) | <0.001 |

**Supplementary Table 3. Sex-covariate interaction testing to determine the impact of female sex on hospitalization in**

**the 6 months before readmission with transthyretin amyloid cardiomyopathy**

| **Additional interaction term** | **Odds ratio for interaction term (95%)** | **p-value** | **Odds ratio for female (95%)** | **p-value** |
| --- | --- | --- | --- | --- |
| No interaction term | **-** | **-** | 1.39 (1.14-1.69) | 0.001 |
| Female#age | 1.00 (0.98-1.01) | 0.72 | 1.68 (0.56-5.04) | 0.35 |
| Female#hypertension | 1.37 (0.75-2.52) | 0.31 | 1.05 (0.59-1.86) | 0.87 |
| Female#diabetes mellitus | 1.25 (0.83-1.88) | 0.28 | 1.29 (1.01-1.64) | 0.042 |
| Female#obesity | 0.49 (0.28-0.84) | 0.009 | 1.55 (1.25-1.92) | <0.001 |
| Female#hypercholesterolemia | 0.98 (0.67-1.44) | 0.92 | 1.40 (1.06-1.86) | 0.018 |
| Female#chronic kidney disease | 1.01 (0.68-1.48) | 0.97 | 1.38 (1.04-1.84) | 0.026 |
| Female#chronic lung disease | 1.33 (1.06-1.66) | 0.014 | 1.21 (0.77-1.90) | 0.40 |
| Female#palliative care | 1.39 (1.14-1.69) | 0.001 | 1.03 (0.28-3.73) | 0.96 |

**Supplementary Table 4: Sex-covariate interaction testing to determine the impact of female sex on the association of**

**prior 6-month hospitalization and in-hospital mortality**

| **Additional interaction term** | **Odds ratio for interaction term for in-hospital mortality (95%)** | **p-value** | **Odds ratio for prior admission and in-hospital mortality (95%)** | **p-value** |
| --- | --- | --- | --- | --- |
| No interaction term | - | - | 2.06 (1.32-3.20) | 0.001 |
| Female#age | 0.99 (0.96-1.02) | 0.62 | 2.06 (1.32-3.20) | 0.001 |
| Female#hypertension | 1.27 (0.42-3.83) | 0.67 | 2.05 (1.32-3.19) | 0.001 |
| Female#diabetes mellitus | 1.52 (0.63-3.67) | 0.36 | 2.05 (1.32-3.18) | 0.001 |
| Female#obesity | 1.45 (0.43-4.84) | 0.55 | 2.07 (1.33-3.22) | 0.001 |
| Female#hypercholesterolemia | 0.91 (0.38-2.18) | 0.84 | 2.06 (1.32-3.20) | 0.001 |
| Female#chronic kidney disease | 1.37 (0.58-3.27) | 0.48 | 2.07 (1.33-3.21) | 0.001 |
| Female#chronic lung disease | 0.99 (0.36-2.75) | 0.99 | 2.06 (1.32-3.20) | 0.001 |
| Female#palliative care | 0.41 (0.12-1.40) | 0.16 | 2.06 (1.33-3.21) | 0.001 |
| Female#liver failure | 2.79 (0.32-24.47) | 0.35 | 2.08 (1.34-3.24) | 0.001 |
| Female#cancer | 1.89 (0.50-7.10) | 0.35 | 2.06 (1.32-3.20) | 0.001 |
